# Endoscopic submucosal dissection of polypoid solitary rectal ulcer

**DOI:** 10.1055/a-2854-6463

**Published:** 2026-05-05

**Authors:** Ahmed Altonbary, Asmaa Gameel, Hazem Hakim, Khadiga Ali

**Affiliations:** 1 Department of Gastroenterology and Hepatology, Mansoura Specialized Medical Hospital 68779 Mansoura UniversityMansouraEgypt; 2 Department of PathologyMansoura UniversityMansouraEgypt

Solitary rectal ulcer syndrome (SRUS) is a medical misnomer because it includes different types of lesions, including mucosal erythema (E-SRUS), ulcers (U-SRUS), polypoid lesions (P-SRUS), and obstructive forms (O-SRUS). Consequently, the endoscopic appearance of the disease is often misdiagnosed as rectal cancer or inflammatory bowel disease 1 . Epidemiological studies have reported that an approximate prevalence of SRUS is one in 100,000 people/y 2 . Surgical repair is the conventional method for the treatment of bleeding P-SRUS 1 . However, the outcome of surgery is usually suboptimal and complications are not uncommon.

**Fig. 1:**
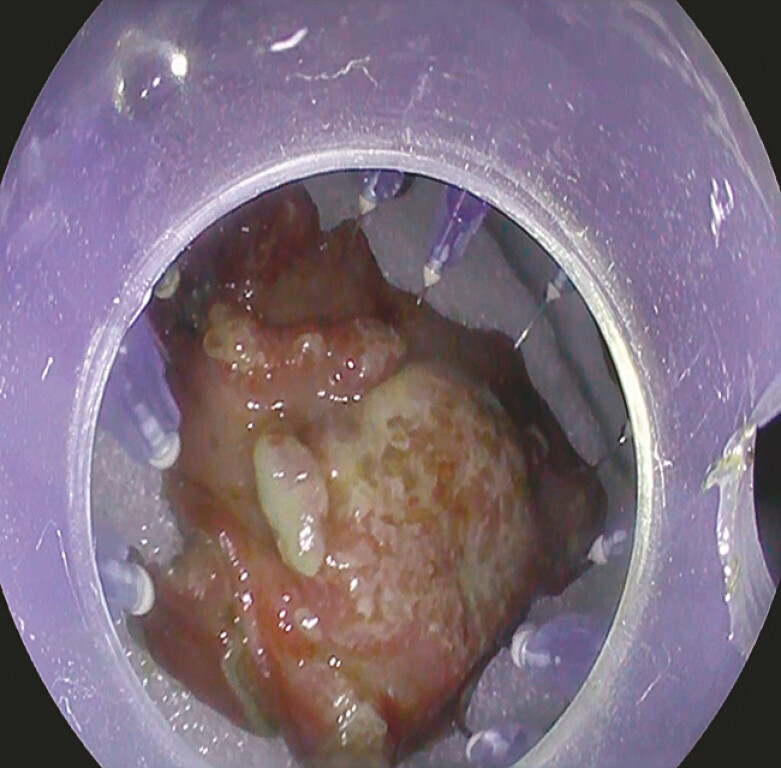
En bloc resection of P-SRUS measuring about 5.5 × 4 cm. P-SRUS, polypoid solitary rectal ulcer syndrome.

**Fig. 2:**
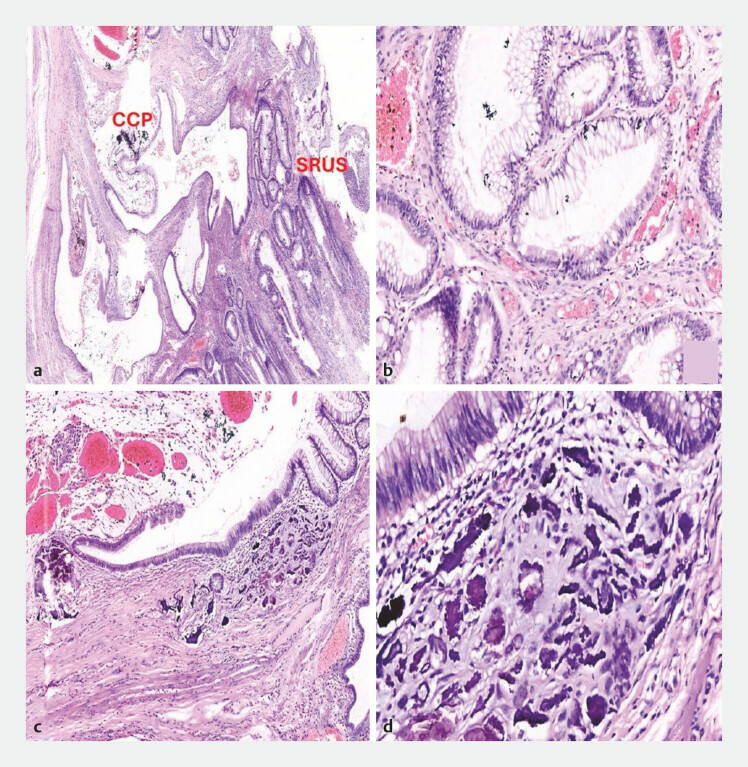
Pathological examination: **a** elongated hyperplastic crypts and thickening of muscularis mucosae (H&E, magnification × 40) and **b** fibromuscular obliteration of lamina propria consistent with solitary rectal ulcer syndrome “SRUS” (H&E, magnification × 200). **c** Submucosal cysts communicating with the lumen through a gap in muscularis mucosae were present indicative of associated colitis cystica profunda “CCP” (H&E, magnification × 40). **d** Features of chronic changes as dystrophic calcification and metaplastic ossification with giant cell reaction were also present (H&E, magnification × 400). SRUS, solitary rectal ulcer syndrome.

A 17-year-old man presented with recurrent bleeding per rectum. Colonoscopy revealed an anorectal polypoid lesion and biopsies revealed hyperplastic changes with low grade dysplasia. His laboratory studies were unremarkable. The patient refused surgical interventions and was planned for endoscopic submucosal dissection (ESD). After initial endoscopic evaluation of the lesion, a 25G needle was used for submucosal injection of normal saline mixed with indigo carmen. Initial incision was made at an anal side with ball type 2 mm knife using Endocut I (2,2,2) followed by the oral side and the edges of the lesion. Submucosal dissection was performed using precise-sect coagulation 4 with extensive fibrosis throughout most of the lesion ( Video 1 ). Careful dissection was performed along the edges of the lesion assisted by gravity traction by changing the patient position when needed till the complete dissection of the lesion en bloc ( Fig. 1 ). After the procedure, the patient was hospitalized for 2 days under intravenous fluids and antibiotics. Pathological examination showed elongated hyperplastic crypts, thickening of the muscularis mucosa (H&E, magnification × 40) and fibromuscular obliteration of the lamina propria consistent with SRUS ( Fig. 2 ). Endoscopic follow up after 3 months showed a healthy scar at the site of previous resection with no recurrent growth. Despite being challenging, ESD could be a safe and effective option for the treatment of symptomatic P-SRUS.

Endoscopic submucosal dissection of polypoid solitary rectal ulcer.Video 1

Endoscopy_UCTN_Code_TTT_1AQ_2AD_3AD
